# Compared to total serum testosterone, calculated free testosterone has a stronger association with lean mass, muscle strength, power, and physical function in older men

**DOI:** 10.1007/s40520-025-03107-3

**Published:** 2025-06-28

**Authors:** Kristian Villars Lolck, Julian Alcazar, Rikke Stefan Kamper, Bryan Haddock, Peter Hovind, Flemming Dela, Charlotte Suetta

**Affiliations:** 1 Department of Geriatric and Palliative Medicine, Copenhagen University Hospital, Bispebjerg and Frederiksberg, Copenhagen, Denmark; 2 CopenAge - Copenhagen Center for Clinical Age Research, Institute of Clinical Medicine, Faculty of Health and Medical Sciences, University of Copenhagen, Copenhagen, Denmark; 3 GENUD Toledo Research Group, Faculty of Sport Sciences, University of Castilla-La Mancha, Toledo, Spain; 4 CIBER on Frailty and Healthy Aging (CIBERFES), Instituto de Salud Carlos III, Madrid, Spain; 5 Department of Clinical Physiology and Nuclear Medicine, Copenhagen University Hospital, Rigshospitalet Glostrup, Copenhagen, Denmark; 6 Department of Clinical Physiology and Nuclear Medicine, Copenhagen University Hospital, Bispebjerg and Frederiksberg, Copenhagen, Denmark; 7 Xlab, Department of Biomedical Sciences, Faculty of Health and Medical Sciences, University of Copenhagen, Copenhagen, Denmark; 8 Sports and Nutrition Research Laboratory, Riga Stradiņš University, Riga, Latvia

**Keywords:** Sarcopenia, Testosterone, Muscle mass, Muscle strength, Muscle power, Physical performance

## Abstract

**Background:**

Low serum testosterone concentrations have been associated with low muscle mass and strength in older men. However, the existing literature is inconclusive.

**Aim:**

To investigate the differences in the relationship between total and calculated free serum testosterone and muscle status, in young vs. old men.

**Methods:**

Body mass index (BMI), fat percentage, appendicular lean mass (ALM), percentage ALM (ALM%), skeletal muscle index (SMI), handgrip strength (HGS), leg extension power (LEP) and 30-s sit-to-stand performance (30-s STS) were measured in 557 healthy Danish men (326 younger (≤ 65 years) and 231 older (> 65 years)) aged 23–92 years. Total serum testosterone, sex hormone binding globulin and albumin were measured (ELISA) and subsequently, calculated free testosterone and free testosterone index (FTI) were computed. A general linear model examined the relationship between testosterone and individual muscle parameters, with age group-interaction, while a pooled effect model examined the relationship between testosterone and a compound of all muscle parameters, adjusted for age, BMI and fat percentage.

**Results:**

Total testosterone was negatively associated with 30-s STS in younger men, and positively associated with LEP in older men. Calculated free testosterone was positively associated with LEP in younger and older men, as well as SMI, ALM%, HGS and 30-s STS in older men. Calculated Free testosterone and FTI, but not total testosterone, were positively associated with the muscle compound in older men.

**Conclusion:**

The present data indicates that calculated free testosterone, compared to total testosterone, is more closely linked to muscle status and physical performance in older men.

**Supplementary Information:**

The online version contains supplementary material available at 10.1007/s40520-025-03107-3.

## Introduction

With the increasing number and proportion of older individuals in the population, there is growing interest in identifying possible interventions to counteract physical decline and impairment in older people. An important factor of age-related physical decline is loss of muscle mass and muscle strength, i.e. sarcopenia [1]. Sarcopenia increases the risk of morbidity, dependency, falls, and mortality in the older population [2]. One of the suggested etiologies to sarcopenia is the age-related decline in anabolic hormones, with a decline of the male sex hormone, testosterone, being a possible contribution to the development of sarcopenia in older men [3], and proposed as a biomarker of sarcopenia in men [4].

Testosterone is known to stimulate protein synthesis in skeletal muscle cells [5, 6] and is an important factor in developing skeletal muscle in men [7]. It likewise stimulates the formation of myogenic precursor cells, i.e. satellite cells and myonuclei [8], thereby increasing muscle mass, the foundation for increased muscle strength [9] and muscle power [10]. In aging men, total serum testosterone concentration declines in a linear pattern [11–13] whereas the decline in the more bioactive free testosterone [11], bioavailable testosterone [13], and the free testosterone index [12] is steeper due to the increase in sex hormone binding globulin (SHBG) with increased age [11–13].

A substantial number of studies have explored the relationship between serum testosterone, lean mass and strength [14–26]. The conclusions are somewhat conflicting, blurring the overall picture of the muscle-testosterone relationship. Some studies have shown a positive relationship between total testosterone and lean mass [14–16] as well as handgrip strength (HGS) [14–18]. Moreover, a positive relation has been shown between free testosterone (calculated free or bioavailable and measured) and both lean mass [15, 16, 19] and HGS [15, 16, 20–22, 27]. However, not all agree on the significant relationship between lean mass/strength and total testosterone [22–24] or free testosterone [13, 25, 26].

Compared to muscle strength, muscle power (the product of force and velocity) is a stronger predictor of physical performance [28, 29], with a steeper age-related decline than HGS and appendicular lean mass (ALM) [30]. Similarly, two studies regarding the relationship between serum testosterone and muscle power found a positive association [21], while the other found no significant association [23]. As such, the serum testosterone-muscle power relationship has yet to be thoroughly explored.

Likewise, literature concerning the relationship between serum testosterone concentrations and physical function is sparse, and the results are inconclusive. In older men, total testosterone concentration has been reported to be positively related to the short physical performance battery (SPPB) score [17], 5 times sit-to-stand (STS) test [23], and maximal gait speed (50-foot walk test) [25]. However, no significant association has been reported in other studies between testosterone concentrations and gait speed [16, 20] or 5-rep STS performance [16, 25, 26]. To our knowledge, the relationship between serum testosterone concentrations and 30-s STS performance has not previously been examined. The 30-s STS test is a commonly used functional test in clinical and research settings, being a variation of the 5-rep STS test which is a powerful predictor of disability [31] and mortality [32]. Compared to the 5-rep STS test, the 30s STS presents a lower flooring (repetitions below 5 are also included) and a higher ceiling effect (it is more physically demanding), and is considered by the EWGSOP2 as a tool to measure muscle strength in diagnosing sarcopenia [1]. In addition, a recent study proved that 30-s STS performance is closely related and translatable to muscle power [33]. In this way, 30-s STS performance is linked to muscle strength, muscle power, and physical performance.

Hence, in the present study, we aimed to investigate the relationship between systemic serum testosterone concentrations (total and calculated free), lean mass, muscle strength, muscle power, and physical performance in a cohort of 557 healthy Danish men aged 23–92 years. Furthermore, we aimed to explore the effect of age on these parameters to enhance our understanding of the potential effect of testosterone on sarcopenia in older men, elucidating inconsistencies among earlier studies and discussing the potential role for testosterone as a predictor or even possible treatment target for sarcopenia.

## Methods

### Study population

Data and subjects for this study were obtained as a secondary analysis of “The Copenhagen Sarcopenia Study”, a population-based cross-sectional study of 1308 healthy men and women conducted at Copenhagen University Hospital Rigshospitalet-Glostrup from December 2013 to June 2016,based on a subpopulation of 3000 healthy men and women aged 20–93 [30], invited from the Copenhagen City heart Study, a prospective cardiovascular cohort study, based on more than 24,000 participants, randomly chosen through population registers, with details of recruitment and characteristics described elsewhere [34, 35].

Data from the male participants (576 men aged 23–92 years) were included and subsequently divided into two subgroups: younger men (YM; *n* = 326, aged ≤ 65 years old) and older men (OM; *n* = 231, aged > 65 years). All men were community-dwelling and characterized as apparently healthy with no acute medical illness, no surgery performed within the last three months, no history of compromised ambulation or prolonged immobilization, and no ongoing usage of medication known to affect body composition. No exclusion based on high/low BMI was made, and no data on comorbidities were collected. No data on race was collected, but the Danish background population is considered predominantly Caucasian. All the included subjects gave written consent, and the Ethical Committee of Copenhagen approved the study (H-3-2013-124).

### Anthropometrics and body composition

Body mass index (BMI) was calculated (body mass·height^− 2^; kg·m^− 2^). Height (m) was assessed without shoes to the nearest 0.1 cm. Body mass (kg) was measured wearing light clothing (hospital shirt) to the nearest 0.1 kg. Body composition was measured by dual x-ray absorptiometry (DXA) (iDXA, GE Lunar, Madison, WI, US) using Encore software version 16.0. ALM was defined as the sum of lean soft tissue from the arms and legs. Skeletal muscle index (SMI; kg·m^− 2^) was computed by normalizing ALM to height (ALM∙h^ـ2^) to account for body size, as previously suggested [36], and secondly, percentage ALM (ALM%) was computed by ALM∙total body weight^− 1^.

### Muscle function and physical performance

Handgrip strength (HGS, kg) was measured using a handheld dynamometer (Jamar, Sammons Preston Rolyan, Chicago, Illinois, USA) with the subject’s arm along the side of the thorax and elbow bent at 90°. Three trials were made for each arm, and the best result was used for further analysis. Leg extension power (LEP, W) was measured using a Nottingham leg extension power rig (University of Nottingham, UK), with the subjects seated with a knee angle of 15° and instructed to push down the footplate, connected to a flywheel at the power rig, as hard and fast as possible, with one leg at a time. The subjects performed two warm-up trials and then at least five trials with 30 s of rest in between until the power produced did not increase in two subsequent trials. The highest power value produced was then used for further analysis.

30-s sit-to-stand performance (30-s STS) was assessed as the maximum (i.e. as fast as possible) number of times a person could rise and sit from a standardized chair within 30 s. The participant was seated in the middle of a standardized chair (with no armrest, seat height 0.45 m), back straight, and arms crossed against the chest [30]. The total number of stands within 30 s was measured. Incorrectly executed stands were not counted.

### Blood samples analyses

Blood samples were drawn from the antecubital vein, on the same days as the other tests, on varying times during the day, and not in a fasting state, due to logistical limitations. The samples were stored in freezers at -80 °C until laboratory analyses were performed. Concentrations of testosterone in serum (total testosterone: SHBG-bound testosterone, albumin-bound testosterone, and unbound testosterone), as well as SHBG, were assessed by electrochemiluminescence immunoassay (Cobas 6000 analytics instrument, Roche, Switzerland). Total testosterone and SHBG were measured using a module e 601. Total testosterone was measured from samples of 20 µL serum. The detection boundaries were 0.87-52.0 nmol·l^− 1^, with intra-assay covariance 2.1–14% and inter-assay covariance 2.5–18.1%. SHBG was measured in samples of 10 µL serum, with detection boundaries of 0.350–200 nmol·l^− 1^, intra-assay covariance 1.-1.7% and inter-assay covariance 1.8–4%. Albumin was measured on Cobas 6000, using a spectrophotometry module c 501 in samples of 2 µL serum, with detection boundaries of 2.0–100 g·l^− 1^, intra-assay covariance 0.7–0.8% and inter-assay covariance 1-1.3%. Two proxy-estimates of free testosterone were computed: Calculated free testosterone was computed according to the method described by Vermeulen et al. [37];

$$\:\left[free\:T\right]=\frac{(\left[total\:T\right]-(N \cdot\:\left[free\:T\right]\left)\right)}{\left({K}_{s}total\:T\right\{\left[{C}_{shbg}\right]-\left[total\:T\right]+{N}_{1}\left[free\:T\right]\left\}\right)}$$

Free testosterone index (FTI) was calculated as the ratio between total testosterone (nmol/l) and SHBG (nmol/l) multiplied by 100.

### Statistical analyses

Data were presented as mean ± standard deviation unless otherwise stated. An independent sample t-test explored differences between the means of younger men (≤ 65 years old) and older men (> 65 years old), since a previous study on this specific population has shown a loss of relative leg lean mass from the age of 65, preceded by an attenuated loss of leg extension power from the age of 60 [38]. Linear and quadratic regression models were used and compared to assess the relationship between different testosterone parameters and age, and the one showing the best fit (based on a significant change in the coefficient of determination (R^2^)) was selected. A general linear model was performed to examine the relationship between every hormone parameter and every muscle parameter with four different models. Adjustments for age and BMI were included since age and BMI are major determinants of lean mass, muscle strength, muscle power, testosterone and SHBG. Adjustments for fat percentage was included, since fat mass has a great impact on serum testosterone levels [39]. For each model, the age group was included as a fixed factor to assess the independent association between younger and older subjects. In addition, the analyses were adjusted for age alone, age and BMI or age, BMI and fat percentage to further explore the age-, BMI- and fat percentage-independent association between hormone parameters and muscle parameters. Only the model adjusted for age and BMI, and the model adjusted for age, BMI and fat percentage, was included in the final manuscript. The unadjusted model, and the model only adjusted for age, can be found in the supplementary files (online resource 1). Likewise, figures of the unadjusted relationship between parameters of muscle function and hormones among older men can be found in the supplementary files (online resource 1). Standardized β-coefficients were calculated to see if the hormone parameters positively or negatively affected lean mass, muscle function and physical function parameters. Finally, to assess the overall effect of each hormone parameter on muscle status (compound of ALM, SMI, ALM%, HGS, LEP and 30-s STS), age-, BMI- and fat percentage-adjusted standardized β-coefficients were pooled using specialized software (Comprehensive Meta-Analysis Version 3.3.070 (Biostat Inc., USA)). All statistical analyses were carried out with SPSS, version 25 (SPSS Inc., USA), and the level of significance was set at α = 0.05. A post hoc statistical power analysis can be found in the supplementary files (online resource 1).

## Results

The general characteristics of the study participants are presented in Table 1. Compared to the younger men, the older men were shorter with a higher BMI (both *p* < 0.05). The older men had lower ALM, ALM%, SMI, LEP, HGS, and 30-s STS than the younger men (all *p* < 0.001). In addition, the older men had lower levels of calculated free testosterone, FTI and albumin, and higher levels of SHBG compared to the younger men (all *p* < 0.001), while there was no difference in body mass (*p* = 0.152) and total testosterone (*p* = 0.070).

**Table 1:** Main characteristics (mean ± standard deviation) of the study participants

	Younger men (*n* = 326)	Older men (*n* = 231)	*P* value
Age (years)	46.7±11.8	74.1±6.0	< 0.001
Height (m)	1.81±0.07	1.77±0.07	< 0.001
Body mass (kg)	84.6±12.1	82.9±15.0	0.152
BMI (kg·m^− 2^)	25.7±3.6	26.5±4.2	0.022
ALM (kg)	27.9±3.6	24.5±3.7	< 0.001
ALM% (%)	33.1±3.0	29.7±2.8	< 0.001
SMI (kg·m^− 2^)	8.46±0.94	7.81±0.97	< 0.001
LEP (W)	344.2±90.7	226.2±81.9	< 0.001
HGS (kg)	51.6±8.4	40.4±8.9	< 0.001
30-s STS (reps)	24.6±6.3	16.7±5.7	< 0.001
Total testosterone (nmol·L^− 1^)	17.8±6.2	16.3±6.7	0.070
Calculated free testosterone (nmol·L^− 1^)	0.256±0.072	0.202±0.065	< 0.001
Free testosterone index (FTI)	41.6±13.4	30.0±10.2	< 0.001
Albumin (g·L^− 1^)	40.4±2.8	37.7±2.7	< 0.001
SHBG (nmol·L^− 1^)	45.4±17.3	56.8±23.1	< 0.001

Note. BMI, body mass index. ALM, appendicular lean mass. ALM%, percentage appendicular lean mass. SMI, skeletal muscle index. LEP, leg extension power. 30-s STS, 30-s sit-to-stand performance. SHBG, sex hormone binding globulin

**Fig. 1:**
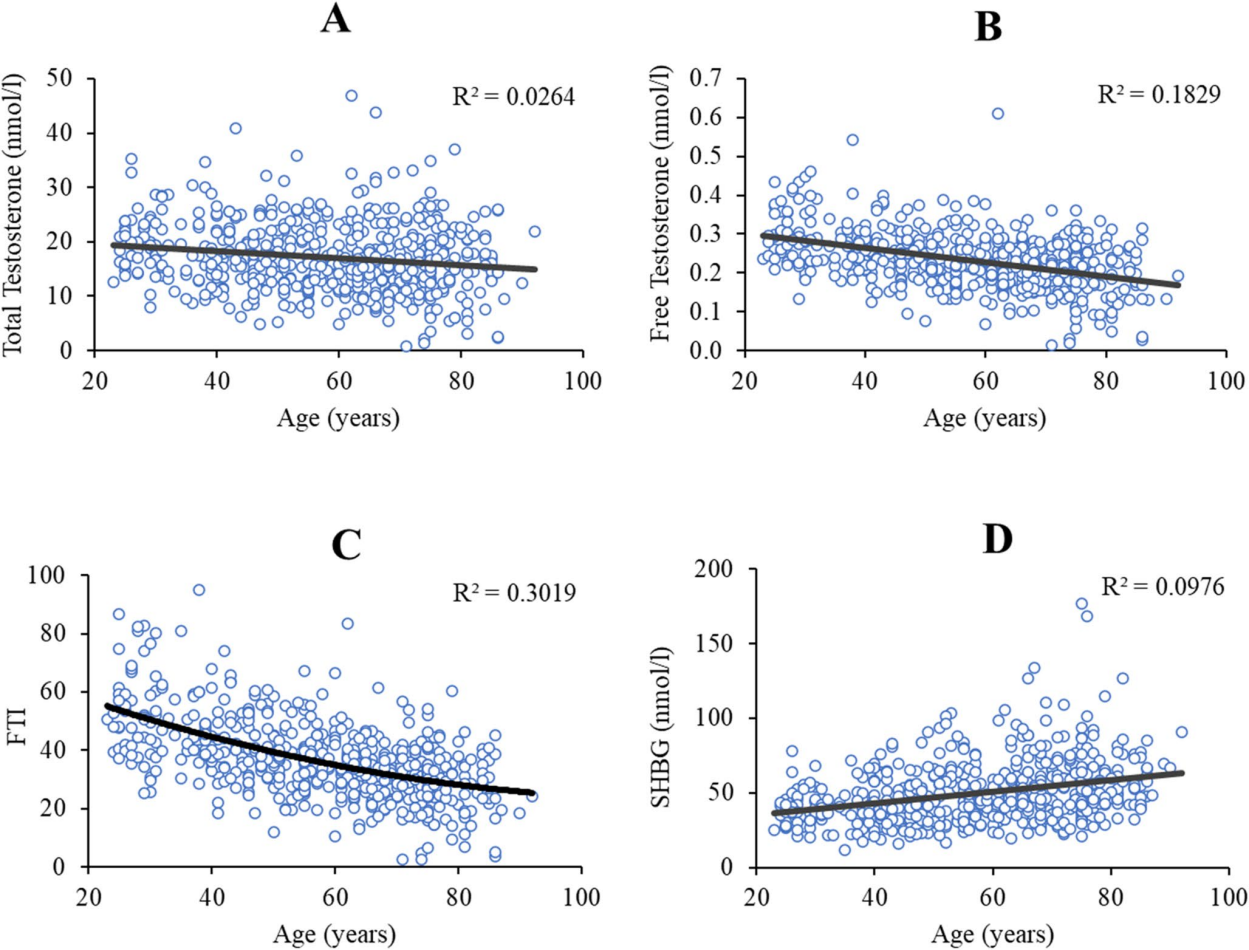
Note. Relationship between age and total testosterone (**A**), calculated free testosterone (**B**), free testosterone index (FTI) (**C**), and sex hormone binding globulin, SHBG (**D**) in a total of 557 healthy men

The associations between age and testosterone parameters are shown in Fig. 1. Both total and calculated free testosterone declined significantly with age in a linear fashion (–0.162 nmol·L^− 1^·year^− 1^ and − 0.428 nmol·L^− 1^·year^− 1^, respectively; both *p* < 0.001), while FTI declined with age in a curvilinear fashion (*p* < 0.001), with the steepest decline occurring at younger ages. Finally, SHBG was found to increase linearly with age (0.312 nmol·L^− 1^·year^− 1^; *p* < 0.001).

Associations between systemic serum testosterone (total, free and FTI) and lean mass, muscle function, and physical performance (adjusted for age and BMI, and adjusted for age, BMI and fat percentage) are shown for younger and older men in Table 2.

**Table 2:** Relationship between hormone levels and the different muscle parameters by age group

		Models			
		*Adjusted for age and BMI*		*Adjusted for Age*,* BMI and FP*	
		Std-β	*p*-value	Std-β	*p*-value
***Total Testosterone***					
ALM	YM	**0.096**	**0.030**	0.040	0.330
	OM	0.033	0.536	-0.024	0.611
SMI	YM	0.066	0.068	-0.020	0.491
	OM	**0.118**	**0.006**	-0.042	0.183
ALM%	YM	**0.113**	**0.008**	0.006	0.802
	OM	**0.123**	**0.025**	0.017	0.628
HGS	YM	0.069	0.226	0.021	0.701
	OM	**0.163**	**0.011**	0.120	0.055
LEP	YM	0.084	0.082	0.067	0.186
	OM	**0.171**	**0.009**	**0.140**	**0.032**
STS	YM	-0.072	0.158*	**-0.111**	0.030*
	OM	0.115	0.089*	0.074	0.263*
***Calculated Free Testosterone***					
ALM	YM	0.068	0.132	0.040	0.113
	OM	**0.104**	**0.039**	0.072	0.113
SMI	YM	**0.080**	**0.032**	0.035	0.179
	OM	**0.144**	**< 0.001**	**0.100**	**< 0.001**
ALM%	YM	**0.093**	**0.035**	0.036	0.203
	OM	**0.158**	**0.003**	**0.099**	**0.003**
HGS	YM	0.069	0.234*	0.039	0.491*
	OM	**0.225**	< 0.001*	**0.199**	< 0.001*
LEP	YM	**0.125**	**0.014**	**0.119**	**0.019**
	OM	**0.273**	**< 0.001**	**0.250**	**< 0.001**
STS	YM	-0.009	0.858*	-0.020	0.702*
	OM	**0.219**	< 0.001*	**0.188**	0.004*
***Free Testosterone Index***					
ALM	YM	0.023	0.631	0.019	0.640
	OM	**0.107**	**0.03**	0.088	0.054
SMI	YM	0.073	0.063	**0.058**	**0.018**
	OM	**0.137**	**0.001**	**0.105**	**< 0.001**
ALM%	YM	0.055	0.248	0.041	0.114*
	OM	**0.155**	**0.004**	**0.115**	< 0.001*
HGS	YM	0.071	0.241*	0.061	0.303*
	OM	**0.230**	< 0.001*	**0.212**	< 0.001*
LEP	YM	**0.120**	0.023*	**0.125**	0.017*
	OM	**0.277**	< 0.001*	**0.259**	< 0.001*
STS	YM	0.035	0.528*	0.041	0.431*
	OM	**0.231**	< 0.001*	**0.204**	0.002*

Note. YM, young men. OM, older men. Std-β, standardized β-coefficients. BMI, body mass index. FP, fat percentage. ALM, appendicular lean mass. ALM%, percentage appendicular lean mass. SMI, skeletal muscle index. HGS, hand grip strength. LEP, leg extension power. STS, 30-s sit-to-stand performance. Bold values indicate *p* < 0.05. *Significant age-by-hormone interaction, denoting significant differences between age groups (*p* ≤ 0.05)

Among younger men, total testosterone was positively associated with ALM% and SMI in the age- and BMI-adjusted model (both *p* < 0.05). After additional adjustment for fat percentage, only 30-s STS performance was associated to total testosterone, with a negative relationship (*p* = 0.03). Calculated free testosterone was positively associated with SMI, ALM%, and LEP the model adjusted for age and BMI (all *p* < 0.05), while only the association LEP remained statistically significant after adjusting for age, BMI and fat percentage(*p* = 0.019). Finally, FTI was positively associated with only LEP in the model adjusted for age and BMI (*p* = 0.023), while both ALM% and LEP were significantly associated with FTI when adjusting for age, BMI and fat percentage (both *p* < 0.05).

In older men, total testosterone was positively associated with SMI, ALM% and 30-s STS performance in the model adjusted for age and BMI (all *p* < 0.05). In contrast, only LEP was positively associated with total testosterone after adjusting for age, BMI and fat percentage (*p* = 0.032). Moreover, calculated free testosterone and FTI were both positively associated all the muscle parameters in the model adjusted for age and BMI (all *p* < 0.05). After adjusting for age, BMI and fat percentage, both calculated free testosterone and FTI were positively associated with SMI, ALM%, HGS, LEP, and 30-s STS performance (all *p* < 0.005).

When adjusting for age and BMI, the hormone-age group interaction was significant for total testosterone and 30-s STS performance (*p* = 0.022), calculated free testosterone and HGS (*p* = 0.031) and 30-s STS performance (*p* = 0.006) and FTI and HGS (*p* = 0.013), LEP (*p* = 0.017) and 30-s STS performance (*p* = 0.010). When adjusting for age, BMI, and fat percentage, the hormone-age group interaction was significant for total testosterone and 30-s STS performance (*p* = 0.017), calculated free testosterone, HGS (*p* = 0.028) and LEP (*p* = 0.011), and FTI and ALM% (*p* = 0.043), HGS (*p* = 0.017), LEP (*p* = 0.036) and 30-s STS (0.028).

**Fig. 2:**
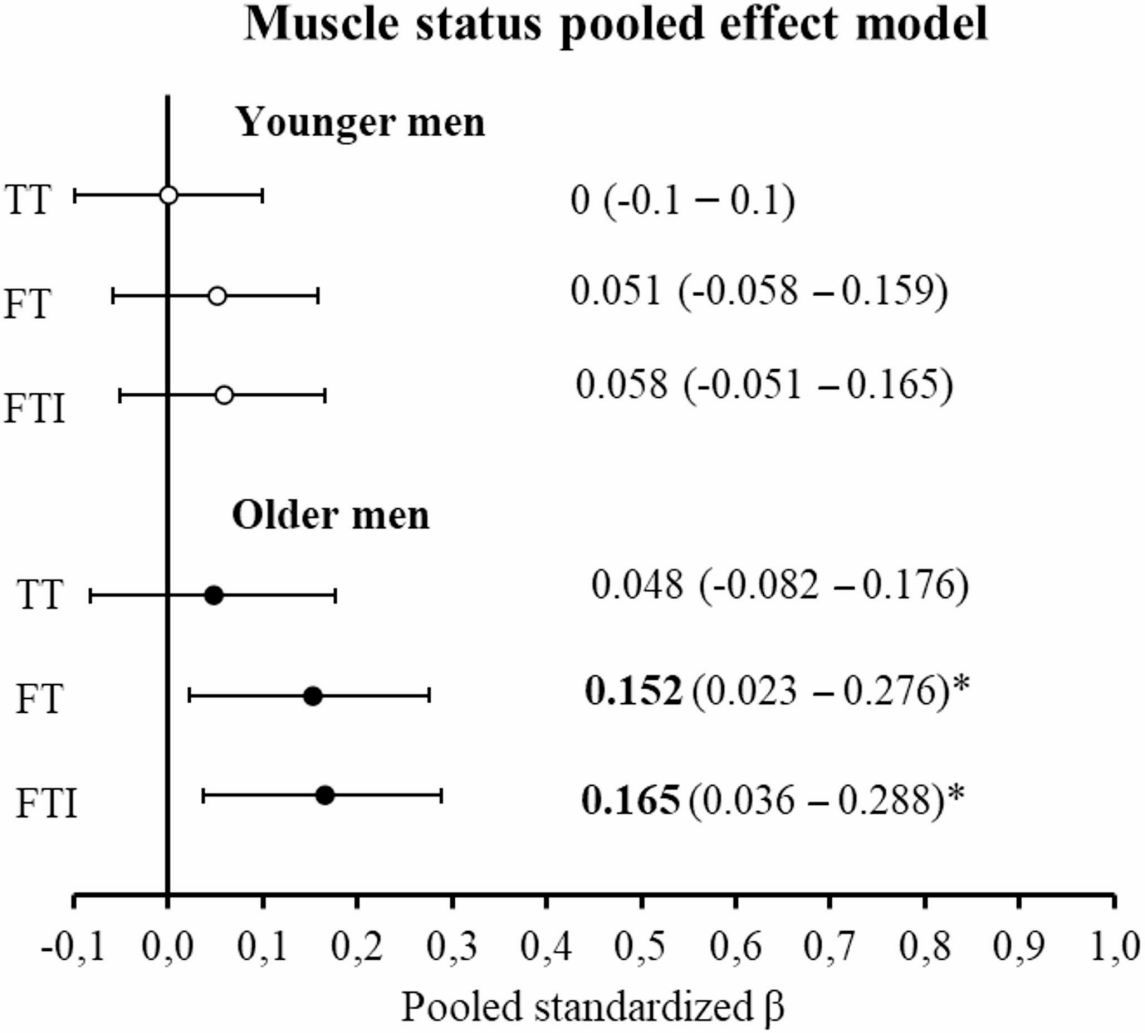
Note. Pooled effect of each hormone parameter (total testosterone (TT), calculated free testosterone (FT), free testosterone index (FTI)) on muscle status (compound of appendicular lean mass (ALM), skeletal muscle index (SMI), percentage ALM (ALM%), handgrip strength (HGS), leg extension power (LEP), and 30-s sit-to-stand performance (30-s STS)) in younger men (≤ 65 years, open symbols) and older men (> 65 years, closed symbols). * *p*-value < 0.05

The overall effect of each hormone parameter on muscle status, adjusted for age, BMI and fat percentage, is shown in Fig. 2. No significant effects were found in younger men. Among older men, muscle status was positively associated with calculated free testosterone (pooled β-coefficient = 0.152, *p* = 0.02) and FTI (pooled β-coefficient = 0.165, *p* = 0.013), but not with total testosterone.

## Discussion

In the present study, we investigated the relationship between systemic concentrations of serum testosterone and appendicular lean mass, muscle strength, power, and physical performance in a population of 557 healthy Danish men aged 23–92 years. The main findings were that after adjusting for age, BMI and fat percentage, total testosterone concentration was negatively correlated with 30-s STS performance in younger but not older men, with significant different age group interaction for 30- s STS performance, and positively associated to LEP only in the older men In contrast, calculated free testosterone concentration was positively correlated to LEP in both younger and older men, as well as with SMI, ALM%, HGS and 30-s STS performance in older men only, with significant different age group interactions for HGS and 30-s STS performance. These findings indicate that calculated free testosterone concentration may be a more clinically relevant indicator of skeletal muscle status than total testosterone concentration, especially in older men.

## Relationship between testosterone and lean mass

Different conclusions can be found in the literature regarding the relationship between testosterone levels and lean mass, with some studies showing a significant positive relationship [14–21], while others did not find any relationship [22–26]. Importantly, the present study showed contrasting results depending on the parameter utilized to determine testosterone levels (total vs. calculated free vs. FTI), the parameter used to determine lean mass (ALM vs. ALM% vs. SMI), the age of the participants (> 65 vs. ≤ 65 years old) and the adjusting variables (age- and BMI-adjusted vs. age, BMI and fat percentage adjusted). This may explain to some extent the differing conclusions found in the literature. Findings derived from absolute levels of ALM in cross-sectional studies should be interpreted with caution since body height declines substantially as a function of participants’ age [40, 41]. Thus, it is recommended to use indexed outcomes of lean mass (e.g. SMI). Further, since testosterone concentration declines with age, age-adjusted models were used to interpret the relationship between testosterone and lean mass. After adjusting for age and BMI, we observed a positive relationship between total testosterone and ALM and ALM% in younger men and SMI and ALM% in older men. Furthermore, both calculated free testosterone and FTI were significantly and positively associated with ALM, SMI and ALM% in older men, independent of age and BMI (Table 2). Auyeung and colleagues [16] found a positive relationship between total testosterone and SMI in older men after adjusting for age, BMI, and SHBG, consistent with the findings of the present study. Interestingly, when adjusting for SHBG, the most important factor is how much total testosterone is in a free state, the authors created a variable similar to FTI (serum testosterone divided with serum SHBG). Of note, we also found an indication that fat mass affects the lean mass-testosterone relationship. Obesity is a well-known down-regulator of testosterone levels [42] but at the same time obese people tend to present higher levels of lean mass than people of normal weight [19]; thus, controlling for fat percentage can reduce the cofounding effect of obesity in the testosterone-lean relationship. After additional adjustments for fat percentage, none of the lean mass parameters was significantly associated to total testosterone or calculated free testosterone, with only SMI to FTI in the younger men. In stark contrast, SMI and ALM% were positively associated to calculated free testosterone and FTI, but not to total testosterone in the older men. This strongly indicates that the concentration of calculated free testosterone and the FTI are more suitable biomarker of lean mass in men older than 65 years, which should be considered when designing future studies exploring the lean mass-testosterone relationship.

### Relationship between testosterone, muscle function, and physical performance

Muscle strength and power have been reported to be more clinically and functionally relevant for middle-aged and older people than muscle mass [43, 44]. As with testosterone levels, muscle strength and power have been reported to decline with age, even earlier and faster than the decline in muscle mass [30, 38]. In contrast, the relationship between BMI and muscle function is not linear, since very high BMI is inversely associated with muscle function [45], which is also the case for very low BMI [46]. Therefore, the age- and BMI-adjusted models utilized in the present study might be adequate in the investigation of the testosterone-muscle function relationship. Accordingly, we observed no relationship between total testosterone and muscle function (strength or power) in younger men after adjusting for age and BMI. Contrasting results were observed in older men regarding total testosterone, with HGS and LEP being positively associated when adjusted for age and BMI, and LEP remaining statistically significant when also adjusting for fat percentage. Calculated free testosterone and FTI were both positively associated with muscle power (LEP) independently of age, BMI and fat percentage in the younger men, but associated to both HGS and LEP in the older men. (Table 2). In agreement with these results, several studies [22–24] in the past have reported no relationship between total testosterone and HGS, and a positive relationship between free or bioavailable testosterone and HGS [15, 16, 20–22], persisting even after adjustments for muscle mass [27], as well as between free testosterone and LEP [21]. Conversely, Baumgartner and colleagues [14] discovered a positive relationship between total testosterone and HGS in older men, although the analysis was not adjusted for age, and Hsu and colleagues [15] observed a positive relationship between total testosterone and HGS in older men, even after adjusting for age and BMI. Currently, existing literature suggests that free or bioavailable testosterone might be a more clinically relevant biomarker for muscle strength and power than total testosterone, especially in older people.

As a measure of physical performance, greater 30-s STS performance showed positive correlations to total testosterone, FTI, and calculated free testosterone in older men. In contrast, among younger men, total testosterone was significantly negatively associated with 30-s STS performance after adjusting for age, BMI and fat percentage. This runs contrary to the hypothesis of a positive relationship between testosterone and physical performance, but might be explained by the fact, that taller persons perform worse in sit-to-stand tests compared to shorter persons, due to biomechanical limitations [47], and shorter men present lower levels of serum testosterone [48]. To our knowledge, no previous studies have explored the relation between 30-s STS performance and serum testosterone, although LeBlanc and colleagues [23] observed, that higher levels of total testosterone at baseline were associated with a lower decline in 5-rep STS performance after a follow-up. Since 30-s STS performance is not only linked to physical performance, but also muscle strength [1] and muscle power [33], it is a valuable predictor of muscle status, and warrants further exploration in future research regarding the muscle-testosterone relationship in older men.

### Summary

The present study strongly indicates that among older men, calculated free testosterone and FTI are stronger associated to muscle function and physical performance (SMI, ALM%, HGS, LEP, and 30-s STS) than total testosterone, due to being more consistently associated with muscle parameters. Both physical performance, muscle function, and lean mass parameters as a compound, termed muscle status, are predicted by calculated free testosterone and FTI, but not total testosterone in older men, as presented in Fig. 2. To our knowledge, no previous study has presented the effect of testosterone on muscle status in a pooled effect model.

The superiority of calculated free testosterone as a predictor of muscle status might be especially important in studies investigating the effect of testosterone supplementation on muscle mass and function in older men, where participants are often included or sub-grouped according to low or high levels of testosterone, in a number of previous studies only on total testosterone [5, 49–52] and not free testosterone. Calculated free testosterone might also be relevant when screening for sarcopenia before exercise interventions in certain frail male patient groups, e.g. fallers or in a rehabilitation setting. Since the observed statistically significant std-beta coefficients are at the most of near-moderate impact (0.99 − 0.259), the use of calculated free testosterone as a potential biomarker might be even more effectful when used in combination with other established biomarkers for sarcopenia, e.g. GDF-15 [53, 54]. 

### Limitations

Our study is cross-sectional in design, and though a positive relationship between testosterone and muscle parameters in older men is shown, causality cannot be determined from the present results. Prospective cohort studies should be designed in the future to establish the causal relationship between testosterone and skeletal muscle mass, muscle function and physical performance in older men. Due to logistic limitations, blood samples were obtained in a non-fasting state, which is in contrast to existing guidelines [55], and not solely in the morning, as suggested by earlier studies [56]. Although this may obscure the age-related differences between young and older men in testosterone levels [56], it should not affect the relationship between muscle and testosterone in older men. Secondly, we used immune assay to measure serum testosterone levels, which is not considered gold standard, but regarded as and accepted alternative to liquid chromatography-tandem mass spectrometry [57]. The method used for calculating free testosterone (Vermeulen method) differs from the gold standard of direct measurement by equilibrium dialysis. Nonetheless, the Danish Endocrinology Society, the European Endocrine Society (ESE) and the Endocrine Society (USA) suggest estimating free testosterone by the Vermeulen method when equilibrium dialysis is not feasible due to high expenses and low availability to clinicians [55, 57–59]. As such, we would argue that by using the Vermeulen method, the conclusions from this study are more relevant for clinicians in a practical setting, as well as for its implementation in clinical screening and treatment strategies. Lastly, information about physical activity, lifestyle, diet, medications, and comorbidities with impact on testosterone concentrations and lean mass and function were not obtained, which would have been beneficial to include as potential confounders. We do point out, that participants were considered apparently healthy, with no acute medical illness, surgery within 3 months, and living independently.

## Conclusion

The present findings showed that calculated free testosterone is stronger associated to lean mass, strength, power, and physical performance among older men, compared to total testosterone. Moreover, we observed that the FTI and calculated free testosterone demonstrated the same relationship with lean mass, muscle function and physical performance among older men.

## Electronic supplementary material

Below is the link to the electronic supplementary material.

Supplementary Material 1

## Data Availability

The data used in the present study are available from the corresponding author [K Lolck] upon reasonable request.
