# Supplementary material for: Compared to total serum testosterone, calculated free testosterone has a stronger association with lean mass, muscle strength, power, and physical function in older men
Source: Aging Clin Exp Res. 2025 Jun 28;37(1):203. doi: 10.1007/s40520-025-03107-3 (PMC12206171; doi:10.1007/s40520-025-03107-3)
Supplement: Supplementary file 1 — Supplementary Material 1 [file 40520_2025_3107_MOESM1_ESM.pdf]

Supplementary information:

Title:

Compared to total serum testosterone, calculated free testosterone has a stronger association with lean mass, muscle strength, power, and physical function in older men.

Journal name:

Aging Clinical and Experimental Research

Author names

Kristian Villars Lolck, Julian Alcazar, Rikke Stefan Kamper, Bryan Haddock, Peter Hovind, Flemming Dela, Charlotte Suetta

Corresponding author:

Kristian Villars Lolck. Department of Geriatric and Palliative Medicine, Copenhagen University Hospital of Bispebjerg and Frederiksberg, Bispebjerg Bakke 23, 2400 Copenhagen NV, Denmark. kristian.kaltoft.lolck@regionh.dk. Orcid-ID 0009-0003-4297-2449

Table of contents

Supplementary table 1

Supplementary figure 1

Post-hoc stastistical power analysis

Supplementary table 1. Relationship between hormone levels and the different muscle parameters by age group.

		Models							
		Unadjusted		+Age		+Age, BMI		+Age, BMI, FP	
		Std-β	p-value	Std-β	p-value	Std-β	p-value	Std-β	p-value
Total Testosterone									
ALM	YM	0.053	0.337	-0.106	0.081	**0.096**	**0.030**	0.040	0.330
	OM	**-0.225**	**<.001**	**-0.232**	**<.001**	0.033	0.536	-0.024	0.611
SMI	YM	**-0.152**	**0.006**	**-0.178**	**0.002**	0.066	0.068	-0.020	0.491
	OM	**-0.215**	**<.001**	**-0.215**	**<.001**	**0.118**	**0.006**	-0.042	0.183
ALM%	YM	**0.317**	**<.001**	**0.257**	**<0.001**	**0.113**	**0.008**	0.006	0.802
	OM	**0.348**	**0.001**	**0.334**	**<.001**	**0.123**	**0.025**	0.017	0.628
HGS	YM	0.046	0.423	0.004	0.944	0.069	0.226	0.021	0.701
	OM	0.086	0.178	0.079	0.207	**0.163**	**0.011**	0.120	0.055
LEP	YM	0.053	0.324	-0.013	0.803	0.084	0.082	0.067	0.186
	OM	0.031	0.666	0.018	0.784	**0.171**	**0.009**	**0.140**	**0.032**
STS	YM	0.036	0.498	-0.026	0.616*	-0.072	0.158*	**-0.111**	**0.030***
	OM	**0.198**	**0.005**	**0.173**	**0.005***	0.115	0.089*	0.074	0.263*
Free Testosterone									
ALM	YM	0.085	0.129	-0.006	0.915	0.068	0.132	0.040	0.113
	OM	0.002	0.981	-0.015	0.811	**0.104**	**0.039**	0.072	0.113
SMI	YM	0.033	0.129	-0.011	0.857	**0.080**	**0.032**	0.035	0.179
	OM	0.002	0.975	-0.006	0.926	**0.144**	**<.001**	**0.100**	**<.001**
ALM%	YM	**0.286**	**<.001**	**0.155**	**0.003**	**0.093**	**0.035**	0.036	0.203
	OM	**0.287**	**<.001**	**0.260**	**<.001**	**0.158**	**0.003**	**0.099**	**0.003**
HGS	YM	**0.135**	**0.017**	0.045	0.435	0.069	0.234*	0.039	0.491*
	OM	**0.208**	**0.001**	**0.191**	**0.002**	**0.225**	**<.001***	**0.199**	**<.001***
LEP	YM	**0.211**	**<.001**	0.079	0.138	**0.125**	**0.014**	**0.119**	**0.019**
	OM	**0.232**	**<.001**	**0.206**	**0.002**	**0.273**	**<.001**	**0.250**	**<.001**
STS	YM	**0.147**	**0.005**	0.012	0.816*	-0.009	0.858*	-0.020	0.702*

	OM	**0.277**	**<.001**	**0.252**	**<.001***	**0.219**	**<.001***	**0.188**	**0.004***
Free Testosterone Index									
ALM	YM	**0.171**	**0.002**	0.068	0.250	0.023	0.631	0.019	0.640
	OM	0.118	0.069	0.099	0.126	**0.107**	**0.03**	0.088	0.054
SMI	YM	**0.161**	**0.003**	**0.131**	**0.029**	0.073	0.063	**0.058**	**0.018**
	OM	0.126	0.052	0.115	0.064	**0.137**	**0.001**	**0.105**	**<.001**
ALM%	YM	**0.205**	**<.001**	0.018	0.753	0.055	0.248	0.041	0.114*
	OM	**0.201**	**0.003**	**0.162**	**0.011**	**0.155**	**0.004**	**0.115**	**<.001***
HGS	YM	**0.190**	**<.001**	0.084	0.168*	0.071	0.241*	0.061	0.303*
	OM	**0.249**	**<.001**	**0.229**	**<.001***	**0.230**	**<.001***	**0.212**	**<.001***
LEP	YM	**0.283**	**<.001**	**0.131**	**0.018***	**0.120**	**0.023***	**0.125**	**0.017***
	OM	**0.303**	**<.001**	**0.271**	**<.001***	**0.277**	**<.001***	**0.259**	**<.001***
STS	YM	**0.120**	**<.001**	0.028	0.609*	0.035	0.528*	0.041	0.431*
	OM	**0.270**	**<.001**	**0.236**	**<.001***	**0.231**	**<.001***	**0.204**	**0.002***

Note. YM, young men. OM, older men. Std-β, standardized β-coefficients. BMI, body mass index. FP, fat percentage. ALM, appendicular lean mass. ALM%, percentage appendicular lean mass. SMI, skeletal muscle index. HGS, hand grip strength. LEP, leg extension power. STS, 30-s sit-to-stand performance. Bold values indicate $p<0.05$. *Significant age-by-hormone interaction, denoting significant differences between age groups ($p \leq 0.05$).

Supplementary figure 1

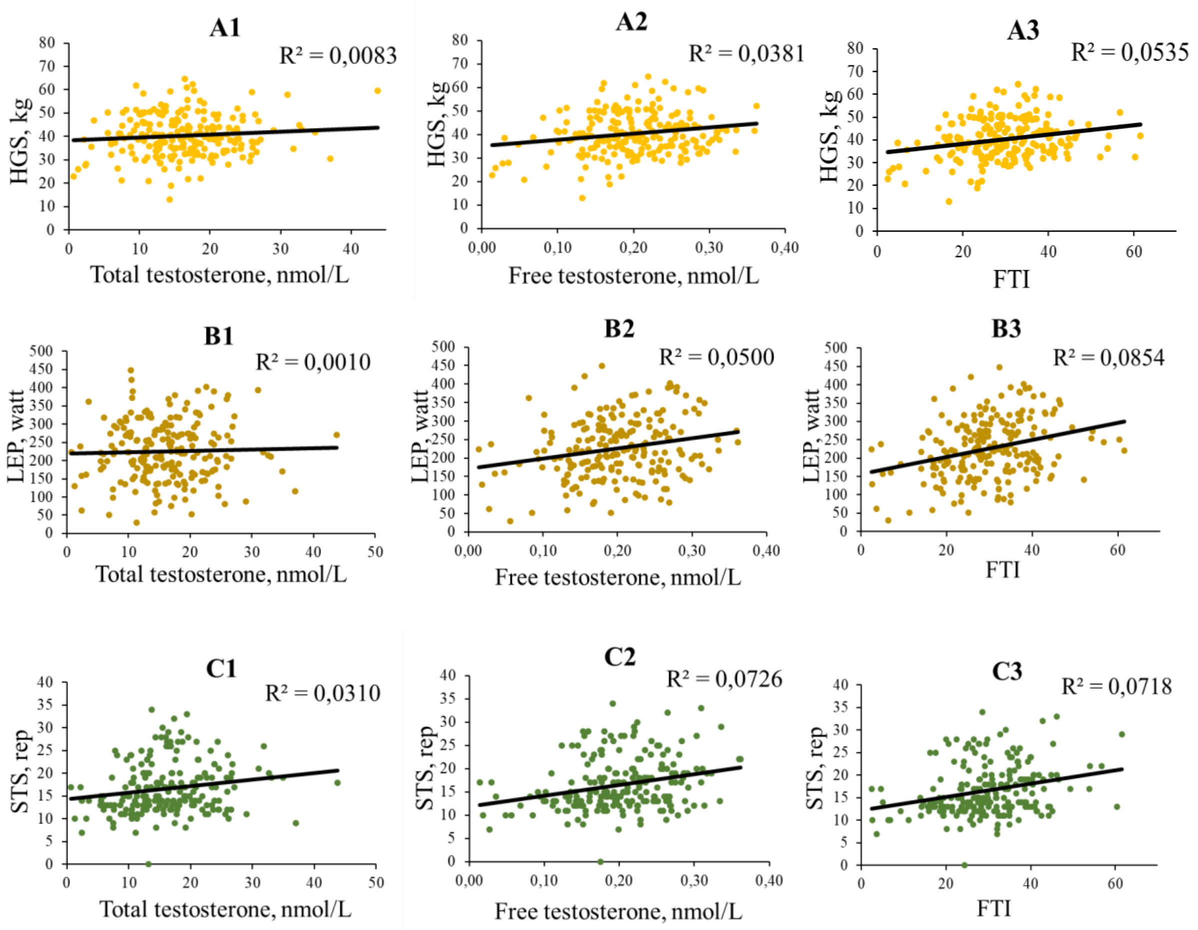

Note. Relationship beteween; handgrip strength (HGS) and total testosterone (A1), free testosterone (A2), and free testosterone index (FTI) (A3); leg extension power (LEP) and total testosterone (B1), free testosterone (B2), and FTI (B3); 30-s sit-to-stand performance (STS) and and total testosterone (C1), free testosterone (C2), and FTI (C3), in older men (age > 65 years, n=231)

Post-hoc stastistical power analysis:

A post hos statistical power analysis was performed free online software (G*power 3.1.9.7, Heinrich-Heine-Universität Düsseldorf). The analysis was performed as a F-test, ANCOVA: Fixed effects, main effects and interactions. Post hoc achieved power was computed using an alpha of 0.05, a sample size of 231 (older men sub cohort), with degrees of freedom set to 6, number of groups to 2, and number of covariates to 4 (Free testosterone, age, BMI, fat percentage). The effect size f was set to 0.55 based on the results from the general linear model (partial eta squared 0.23, older men free testosterone estimates on SMI). This resulted in a power of 0.999.

Subsequently, we lowered the sample size to 52, which resulted in a power of 0.805. In this regard, we consider this study well powered.
